# Recognition of Daily Activities of Two Residents in a Smart Home Based on Time Clustering

**DOI:** 10.3390/s20051457

**Published:** 2020-03-06

**Authors:** Jinghuan Guo, Yiming Li, Mengnan Hou, Shuo Han, Jianxun Ren

**Affiliations:** 1 School of Information Science & Technology, Dalian Maritime University, Dalian 116026, China; liyiming@dlmu.edu.cn (Y.L.); hmn19980810@dlmu.edu.cn (M.H.); hs1120190279@dlmu.edu.cn (S.H.); adsc666@dlmu.edu.cn (J.R.); 2 Artificial Intelligence Key Laboratory of Sichuan Province, Sichuan University of Science and Engineering, Zigong 643000, China

**Keywords:** activity recognition, sensor, smart home

## Abstract

With the development of population aging, the recognition of elderly activity in smart homes has received increasing attention. In recent years, single-resident activity recognition based on smart homes has made great progress. However, few researchers have focused on multi-resident activity recognition. In this paper, we propose a method to recognize two-resident activities based on time clustering. First, to use a de-noising method to extract the feature of the dataset. Second, to cluster the dataset based on the begin time and end time. Finally, to complete activity recognition using a similarity matching method. To test the performance of the method, we used two two-resident datasets provided by Center for Advanced Studies in Adaptive Systems (CASAS). We evaluated our method by comparing it with some common classifiers. The results show that our method has certain improvements in the accuracy, recall, precision, and F-Measure. At the end of the paper, we explain the parameter selection and summarize our method.

## 1. Introduction

The problem of aging in the world’s population is becoming increasingly serious; meaning, the proportion of the aging population is increasing while the fertility rate continues to decrease. By the end of 2019, the number of elderly people worldwide exceeded infants and young children. The problems brought about by an aging population are not only political and financial; in the reality of human aging, brain power will gradually weaken, and symptoms, such as decreased memory and brain function, will occur. The elderly care problem brought about by this is becoming increasingly prominent. Some empty-nest seniors are old and frail, and there is no one to take care of them in emergencies, such as falls, which may cause irreparable losses.

To improve this problem, some researchers have begun to focus on elderly activity recognition in smart homes. Elderly activity recognition is an important part of the functioning of smart homes, mainly as it could determine abnormalities in the elderly by obtaining information about their daily activities. This can help predict some potential diseases in the elderly, such as Alzheimer’s disease, which is also the main motivation for many activity recognition studies in intelligent environments (and has widely been recognized by families affected by Alzheimer’s disease) [1].

According to different data collection methods, smart home activity recognition can be roughly divided into intrusive and non-intrusive design.

Intrusive design usually refers to video-based activity recognition. It mainly records the daily lives of the elderly through cameras, and then methods are used to analyze the video for activity recognition [2,3,4]. The effect of video-based activity recognition is excellent [5], but its disadvantages are also very prominent. First, it collects a lot of privacy and sensitive information. Second, it is susceptible to light. Finally, it is very expensive.

For non-intrusive design, it is mainly divided into wearable and environmental interaction activity recognition. Wearable devices mainly refer to miniature sensors, such as accelerometers and gyroscopes [6,7,8,9], which can be worn on the body. When older people wear them, they can collect information anytime, anywhere. However, wearing these devices can be a burden and cause resentment in the wearers.

With the development of the Internet of things technology [10,11], smart homes have become more complete, making activity recognition popular. This method mainly interacts with seniors by embedding sensors in objects in the smart home. These sensors are mainly divided into environmental sensors and binary sensors. Environmental sensors can collect environmental information in real time. Binary sensors can interact with seniors to locate them. This method is secretive and convenient, and will not cause resentment in the elderly.

In this paper we focus on environment-based interactive activity recognition. Many commonly used methods characterized by statistical sensor frequency ignore the temporal correlation of features. In order to improve the methods, we propose a data-driven activity recognition method. The method is divided into three stages: feature extraction, temporal clustering, and activity recognition. The method of the feature extraction proposes a de-noising method to remove interference from other residents. Generally it is believed that the daily activity of the elderly has a certain regularity, so we can cluster the activities in the close time. In the end, the similarity matching equation proposed in the paper is employed to calculate out the points with the most similarity, and select the classification by the votes of the points.

The structure of this paper is as follows: Section 2 introduces related work. Section 3 defines some terminologies. Section 4 describes our method. In Section 5, we validate our method and discuss it. Section 6 concludes the paper.

## 2. Related Work

Activity recognition based on non-intrusive design can be divided into data-driven and knowledge-driven, according to the differences of the used methods.

The knowledge-driven method extracts things, space, and time into certain rules in the domain, constructs them into a reusable context model and correlates with activities, and then uses inference and other technologies to determine the activity category. Ontology is often used in knowledge-driven methods. Chen et al. [12] proposed a formal explicit ontological modeling and representation of the smart home domain approach to the processing of multisource sensor data streams. Ye et al. [13] proposed a hierarchical structure of the domain concept ontology model to represent domain knowledge, which is independent of particular sensor deployment and activities of interest. Their subsequent researches [14] combined the ontology and statistical methods to automatically detect the boundaries of different activities and extended the Pyramid Match Kernel (PMK) to accommodate and balance the sensor noise in activity recognition. Ontology is also used for cross-environment activity recognition. Wemlinger et al. [15] proposed a Semantic Cross-Environment Activity Recognition (SCEAR) system to establish different ontology models for 22 data sets provided by CASAS to map between different environments. This method has a good recognition effect on two smart homes with similar layouts. The knowledge-driven method can clearly distinguish activities with significant semantic differences, but cannot identify well the activities with similar semantics.

Compared with knowledge-driven, data-driven models place more emphasis on the use of large-scale data for reasoning to build decision models [16]. Data-driven is mainly focused on supervised methods to recognize unlabeled activities by generating classifiers with labeled activities. Ravi et al. [17] proposed a naive Bayesian approach to activity recognition using accelerometers. Ruben et al. [18] proposed a resident adaptation technique based on Hidden Markov Models (HMMs). This system segments and recognizes six different physical activities using inertial signals from a smartphone. Asghari P. et al. [19] proposed an online hierarchical hidden Markov model to predict the activity in the environment with any sensor event. This method first uses the HMM model to recognize the beginning and end of an activity, and then predicts the next activity by establishing an HMM for each sensor event.

Some ensemble methods have also been applied in activity recognition. Hu et al. [20] proposed a novel separating axis theorem (SAT) based splitting strategy, then used it to improve the random forest. Anna et al. [21] used a previously developed Cluster-Based Classifier Ensemble [22] (CBCE) method for smart home-based activity recognition; this method proposes a support formula for clustering to solve the recognition problem in clusters. In addition, with the development of deep learning, neural networks have also been applied to activity recognition. Arifoglu D et al. [23] extracted fixed-length sliding windows into a sparse two-dimensional time matrix to use Convolutional Neural Networks (CNN) for activity recognition. This is basically the same as the work of Gochoo et al. [24], and both have been tested on the Aruba dataset (and the performance is roughly the same). However, Arifoglu et al. proposed a novel method for identifying abnormal activities, and discovered abnormalities, as well as identified and prevented abnormal activities by generating abnormal activities in the data set. Medina et al. [25] proposed a method using fuzzy time windows (FTW) to segment the data set, followed by Long Short-Term Memory (LSTM) for activity recognition.

There are also unsupervised and semi-supervised methods. The semi-supervised method mainly uses a small part of the labeled data to label a large amount of data; thereby, reducing the workload of the labeling activity. Hu et al. [26] proposed a cross-domain activity recognition (CDAR) algorithm to label another set of different but related activity from labeled activities. Wen [27] et al. proposed a similarity measurement formula that uses a small amount of labeled data to label a large amount of unlabeled data. The difficulty of the unsupervised method is the problem of data labeling. Researchers have now proposed some unsupervised methods to solve the problem of data annotation, such as frequent sensor mining methods [28], and frequent periodic pattern mining methods [29], activity modeling based on low-dimensional feature space [30], probabilistic model [31,32], and retrieval of activity definition, using Web mining [33].

Some multi-resident activity recognition based on smart homes has also been proposed. Hao et al. [34] proposed a knowledge-driven solution based on formal concept analysis (FCA) and sequential pattern mining to analyze the activity rules of different residents and recognize human activities in non-intrusive sensor data. Alemdar et al. [35] used the factorial hidden Markov model and nonlinear Bayesian tracking to recognize the behaviors of the two residents. They did not distinguish between the residents and achieved good results. Guo et al. [36] proposed a method to extract features using the improved Term Frequency-Inverse Document Frequency (TF-IDF). They used the improved TF-IDF to calculate the probability of the sensor appearing in the activity, recognized it as a new feature, and tested the method on the Tulum2009 and Cairo datasets. Lu et al. [37] used Back Propagation-Hidden Markov Model (BP-HMM) to extract daily activity features and SVM to recognize daily activity. They introduced the dependent Beta process into the HMM, and integrated the state constraints of the sensors into the sampling process. Finally, SVM is employed to recognize daily activities.

Many multi-resident activity recognition focuses on feature extraction. This paper proposes a clustering method based on the time pattern of elderly activity, then based on this, a similarity matching formula is proposed. This formula is based on Levenshtein Distance which is used in the stage of activity recognition model development, rather than the stage of daily activity feature extraction. We applied this method to the activity recognition of two residents and the results were very attractive. Although the cost of training will be higher, this method is better than a large number of single and ensemble methods.

## 3. Terminologies

To better express our method, we define some terminologies and use the activities in Table 1 as examples.

**Definition** **1.** *se = (d, t, s, ss, ar, as) is a sensor event, where d is the date when the event occurred, t is the time when the event occurred, s is the activated sensor, ss is the sensor status, ar is the corresponding activity, and as is the activity status.*

In this paper we use *se.d*, *se.t*, *se.s*, *se.ss*, *se.ar*, *se.as* to represent *d*, *t*, *s*, *ss*, *ar*, *as* in this event. Table 1 shows the two activities in the dataset and the meaning of *s* = (*d*, *t*, *s*, *ss*, *ar*, *as*). For example, *se*_1_ = (*d*, *t*, *s*, *ss*, *ar*, *as*) represents *se*_1_ = (2010-11-04, 05: 40: 51.303739, M004, ON, Bed_to_Toilet, begin).

**Definition** **2.** *For a given sensor event se_1_, se_2_... se_n_, we connect their s in series, sq = {se_1_.s, se_2_.s ... se_n_.s} is called a sensor sequence, which is simplified to sq = {s_1_, s_2_ ... s_n_}.*

The sensor sequence of Bed_to_Toilet activity in Table 1 is expressed as *sq*_1_ = {M004, M005, M007, M001, M004, M004, M007, M001}, and the sensor sequence of Sleep is expressed as *sq*_2_ = {M004, M004.M007, M007, M006, M007, M005, M004}. Note that we use the part where the activities intersect as common to both activities.

**Definition** **3.** *For activity a and a series of sensor events, we represent the activity as a = {bt, et, sq}, where bt is se_1_.t retention hour, et is se_n_.t retention hour, and sq is the sensor sequence in Definition 2.*

For example, the Bed_to_Toilet activity in Table 1 can represent *a* = {05, 05, *sq*_1_}, where *sq*_1_ = {M004, M005, M007, M001, M004, M004, M007, M001}.

**Definition** **4.** *We count the importance of other sensors in the activities that begin with a sensor and store them in the SIA. The algorithm is shown in Algorithm 1.*

**Algorithm 1:** GettingSIA**Input:***Sq*{*sq*_1_, *sq*_2_,*…sq_n_*}, a set of sensor sequence          *Sensor*, sensor set**Output:***SIA*, two-dimensional tuple of sensor importance1. *SIA*←Ø;2. *fre*←Ø;//*fre* is a key-value map to counts the number of activities begin with a certain sensor3. **for each**
*sq*
**in**
*Sq*:4.    *Arr←*Ø;//*Arr* is a tuple that counts whether a sensor is present 5.    *sv← getT(sq.s*_1_)*+1*//*getT(sq.s*_1_) is employed to get times *t* of *sq.s*_1_6.    *update*(*fre*,{*sq.s*_1_,*sv*})7.    **for each**
*s*
**in**
*sq:*8.        *Arr←*◡{(*sq.s*_1_, *1*)}9.    **end for**10.   *St←getDict(SIA*,*sq.s*_1_)//*getDict(SIA*,*sen*) is employed to get dict *sen*11.   *St←sum(St*, *Arr*)}//sum is add *Arr* to *St**12.*   *update*(*SIA*,{*sq.s*_1_,*St*}) 13. **end for**14. *SIA←div(SIA*, *fre*)//div is used to calculate *SIA* divided by *fre*
15. **return**
*SIA*

As shown in Table 1, there are two sensor sequences starting with M004, namely *sq*_1_ = {M004, M005, M007, M001, M004, M004, M007, M007}, *sq*_2_ = {M004, M004, M007, M007, M006, M007, M005, M004}, then through Algorithm 1 we can get the importance of the sensor of the activities beginning with M004. The result is {((M001,0.5) (M004,1), (M005,1), (M006,0.5), (M007,1)}, which is represented in *SIA* as *SIA*{(M004, {(M001,0.5) (M004,1), (M005,1), (M006,0.5), (M007,1)})}.

## 4. Methodology

**Figure 1:**
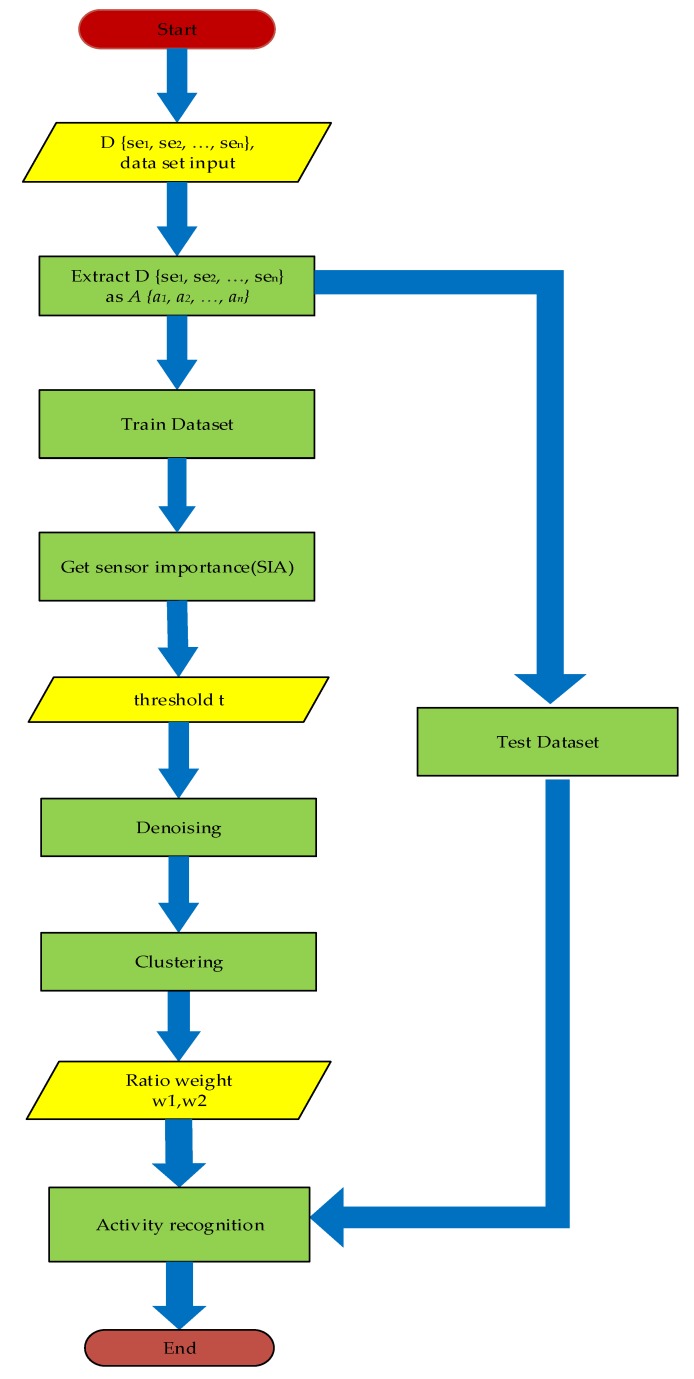
Process for the activity recognition.

Our method is mainly divided into two parts: feature extraction and activity recognition. The process is shown in Figure 1.

### 4.1. Feature Extraction

The feature extraction method is shown in Algorithm 2. First, we extract the data set *D* = {*se*_1_, *se*_2_… *se_n_*} and extract it as *A* = {*a*_1_, *a*_2_… *a_n_*}. Next, we need to de-noise *sq* in *A*. It is believed in a two-residential residence, sensor events triggered by one resident may be disturbed by another resident, and these interferences can be removed by our method.
**Algorithm 2:** Feature Extraction**Input:**  *A*{*a*_1_, *a*_2_*…a_n_*}, a set of activity        *SIA*, a tuple of sensor importance        *w*, threshold**Output:***Ar*{*a*_1_,*a*_2_*…a_n_*}, activities after feature extraction1. *Ar*←Ø; 2. **for each**
*a*
**in**
*A*:3.    *ns*←Ø;//new sensor sequence4.    **for each**
*s*
**in**
*a.sq*:5.       *temp*←*getvalue(SIA*,*sq.s*_1_,*s*)//Get the importance of *s*6.       **if**
*temp* > *w*
**and**
*s does not repeat*:7.          *ns*←◡*s*8.       **end if**9.    **end for**10.   *Ar*←◡{*a.bt,a.et,ns*}11. **end for**12. **return**
*Ar*

Our de-noising method has two steps: the first step is to remove duplicate sensor features. It means that if *sq.s_i_* is equal to *sq.s_i +_*_1_ in *sq*, then only *sq.s_i_* is kept. In the second step, we propose a sensor importance measurement method. As shown in Definition 4, *SIA* counts the importance of other sensors for each sensor that appears at the beginning of the activity. If the importance exceeds a set threshold, we keep the sensor.

Through the above two steps of screening, the extracted feature *Ar* = {*bt*, *et*, *sq*} is finally obtained.

### 4.2. Activity Recognition

Our activity recognition method is to recognize the activities after extracting features. First, we cluster the activities with the Kmeans based on the *bt* and *et* in *ar*. The number of clusters *k* was selected using the elbow method. The core index of the elbow method is *SSE* (sum of the squared errors), the formula is:

(1) $$\mathrm{SSE}=\sum_{i=1}^{k}\sum_{p\in C_{i}}{\left|p-m_{i}\right|}^{2},$$

where *C_i_* is the i-th cluster, *p* is the sample point in *C_i_*, *m_i_* is the centroid of *C_i_* (mean of all samples in *C_i_*), and *SSE* is the clustering error of all samples, which represents the quality of the clustering. The relationship between *SSE* and *k* is used to obtain *k* with the largest curvature in the graph. The specific algorithm is shown in Algorithm 3.
**Algorithm 3:** SelectK**Input:**  *Ar*{*a*_1_,*a*_2_*…a_n_*}, a set of activity        *num*, number of active classes**Output:***k*, Optimal k value1. *Val*←Ø//2. *Cal*←Ø//3. *k*←Ø4. *Time*←*getTime*(*Ar*)//filter out *bt* and *et*5. **for each**
*n*
**in**
*range*(1, *num*):6.    *estimator*←*KMeans*(*clusters*=*n*)//constructing a cluster7.    *estimator.fit*(*Time*)//fitting the data8.    *Val*←*getSSE*(*estimator*)//*get SSE value*9.    *Cal*←◡{(*n*, *Val*)}10. **end for**11. *k*←*getCur(Cal*)//Get k with highest curvature12. **return**
*k*

After clustering, we first find the cluster that belongs to the input test data, and then calculate the similarity between the instances in the cluster and the test data. Here we propose a similarity matching method. If the test case is *t* = {*bt*, *et*, *sq*}, the instance in the training set is *a* = {*bt*, *et*, *sq*}, then the similarity between them is expressed as:

(2) $${\mathrm{ratio}=w}_{1}*\frac{24-\left|a.\mathrm{bt}-t.\mathrm{bt}\right|}{24}{+w}_{1}*\frac{24-\left|a.\mathrm{et}-t.\mathrm{et}\right|}{24}{+w}_{2}*\mathrm{Levenshtein}.\mathrm{ratio}(a.\mathrm{sq},\;t.\mathrm{sq}),$$

where *w*_1_ and *w*_2_ refer to the weight represented by time and sensor sequence. Here we make${\;2*w}_{1}{+w}_{2}=1$, 24 refers to 24 h in a day. Levenshtein.ratio is a method to calculate the similarity of sequences, the formula is:

(3) $$\mathrm{Levenshtein}{.\mathrm{ratio}(\mathrm{sq}}_{1}{,\mathrm{sq}}_{2})=\frac{\mathrm{sum}-\mathrm{ldist}}{\mathrm{sum}},$$

where *sum* refers to the sum of the length of *sq*_1_ and *sq*_2_, and *ldist* is the class edit distance.

After obtaining the ratio, select the *n* closest instances in the cluster and let them vote for the label of test data. The complete recognition algorithm is shown in Algorithm 4.
**Algorithm 4:** Recognize Activity**Input:**  *Ar*{*a*_1_,*a*_2_*…a_n_*}, a set of activity         a_t_, test sample         *w1*,*w2*, weight of ratio         n, select the number of training instances with the highest similarity**Output:***label*, *label of a_t_*1. *Time*←*getTime*(*Ar*)//get bt and et2. *AllRatio*←Ø3. *k*←*SelectK(Ar*)//SelectK is used to select the optimal k4. *clf*←*KMeans*(*n_clusters*=*k*)//clustering5. *clf.fit*(*Time*)//fitting the data6. *m*←*clf.predict(*{*a_t_.bt*,*a_t_.et*})//find the cluster where the test sample is located7. **for each**
*a*
**in**
*Ar:*8.    **if**
*cluster(a*) **==**
*m:*9.       *ratio***←***getRatio(a*,*at*,*w1*,*w2*)//calculate ratio10.       *AllRatio*←◡*ratio*11.    **end if**
12. **end for**13. *sort*(*AllRatio*)//sort max to min14. **for** i **in range**(n):15.    *val*← *getALLRatio(i*)**/**/get the first n instances of maximum ratio16.    *topk_y*←◡*val*17. **end for**
18. *label*←*vote*(*topk_y*)//vote for label19. **return**
*label*

## 5. Results and Evaluation

### 5.1. Datasets

We used two two-resident datasets from Washington State University for experiments [38]; they are “Tulum2010” and “Cairo”. Details of these two datasets are listed in Table 2.

**Figure 2:**
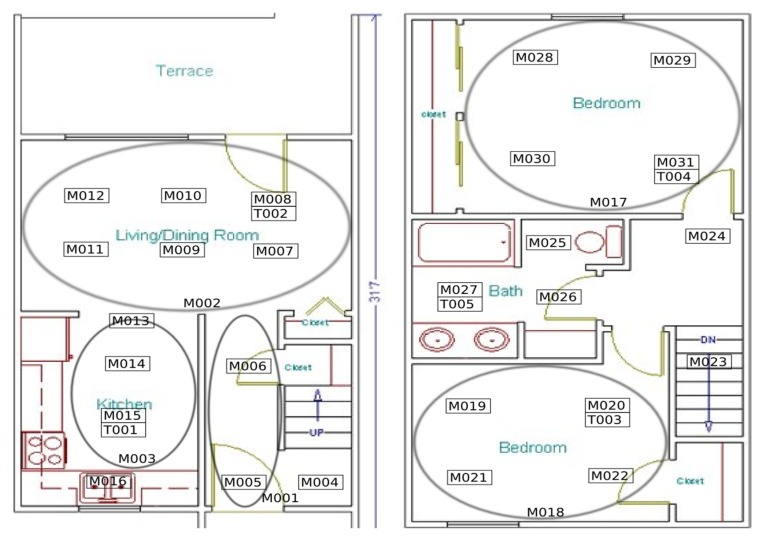
Tulum2010 sensor layout.

The Tulum2010 dataset was collected in the Washington State University (WSU) Tulum smart apartment during 2009–2010. The apartment housed two married residents who performed normal daily activities. The sensor layout is shown in Figure 2. We selected the first three months of the data set.

Tulum2010 dataset contains 31 motion sensors (M001 through M031) and 5 temperature sensors (T001 to T005). Its 14 activities are “Bathing”(B), “Eating”(“E”), “Bed_Toilet_Transition”(“B_T”), “Watch_TV”(“W_TV”), “Work_Table”(“W_T”), “Work_LivingRm”(“W_L”), “Enter_Home”(“E_H”), “Leave_Home”(“L_H”), “Personal_Hygiene”(“P_H”), “Sleeping_in_Bed”(“S_B”), “Yoga”(“Y”), “Work_Bedroom_1” (“W_B1”), “Work_Bedroom_2” (“W_B2”), “Meal_Preparation”(“M_P”).

**Figure 3:**
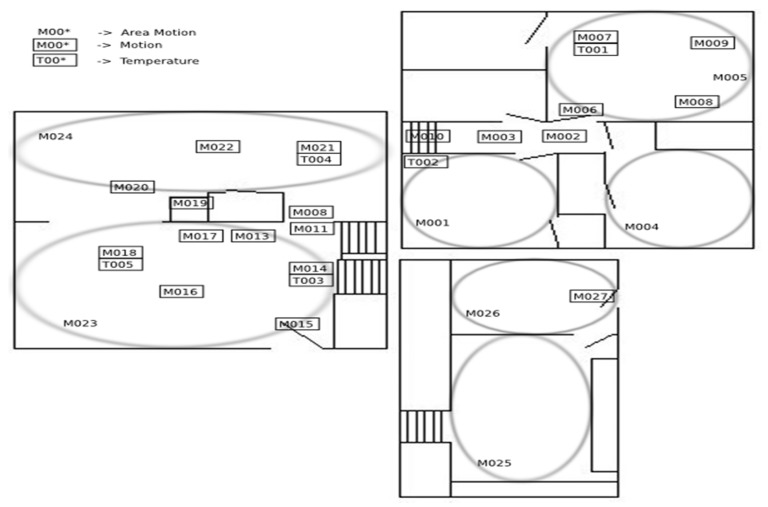
Cairo sensor layout.

The Cairo dataset was collected in the home of a voluntary adult couple. A couple and a dog live in the smart apartment. The couple’s children also visited the house at least once. The sensor layout is shown in Figure 3.

Cairo dataset contains 27 motion sensors (M001 to M027) and 5 temperature sensors (T001 to T005). Its 13 activities are “Bed_to_toilet”(“B_T”), “Breakfast”(“B”), “Night_wandering”(“N_W”), “R2_wake”(“R2_W”), “Leave_home”(“L_H”), “R2_take_medicine”(“T_M”), “R1_sleep”(“R1_S”), “Lunch”(“LUN”), “Laundry”(“L”), “R1_wake”(“R1_W”), “R2_sleep”(“R2_S”), “Dinner”(“D”), “R1_work_in_office”(“W_O”).

### 5.2. Evaluation Method

In this section, we mainly applied all the examples in the considered dataset and performed five cross-validations to evaluate the performance of our method. During the process, it was performed five times. In each iteration, four folds are selected for training and one fold is used for testing. The final accuracy is the average of the five results. Except for the accuracy, in the paper we also used others to evaluate the results, such as the precision, the recall, and the F-Measures. We used the confusion matrix in Table 3 to represent the number of true positives, true negatives, false positives, and false negatives for the 2 classification problems.

The columns and rows in the matrix refer to the actual and predicted classes by a classification model, respectively. Based on the confusion matrix the precision, recall and F-Measure for class 1 can be calculated as presented in Equations (4)–(7), respectively:

(4) $$\mathrm{Accuracy}=\frac{\mathrm{TP}+\mathrm{TN}}{\mathrm{TP}+\mathrm{FP}+\mathrm{FN}+\mathrm{TN}},$$

(5) $$\mathrm{Precision}=\frac{\mathrm{TP}}{\mathrm{TP}+\mathrm{FP}},$$

(6) $$\mathrm{Recall}=\frac{\mathrm{TP}}{\mathrm{TP}+\mathrm{FN}}$$

(7) $$F-\mathrm{Measure}=\frac{2*\mathrm{TP}}{2*\mathrm{TP}+\mathrm{FP}+\mathrm{FN}}$$

The four measures were calculated for each class, taking into account the class imbalance problem; the final result is presented as the average of all classes.

### 5.3. Results and Evaluation

We first compare our results with several common single classifiers, such as K-Nearest Neighbor (KNN), lib Support Vector Machine (libSVM), Sequential Minimal Optimization (SMO), Naïve Bayes (NB), PIPPER, C4.5 and Random Forests (RF). In addition to these, we also compare several more complex classifiers. These classifiers are all implemented in Weka, and the specific results are shown in Table 4 and Table 5.

It can be seen from Table 4 and Table 5 that our method and RF are the highest performance methods, and our method is better than RF in terms of performance. To better understand the performance of our method, we construct a confusion matrix. Table 6 and Table 7 show the confusion matrices for Tulum2010 and Cairo.

From Table 6, we can see that in the Tulum2010 dataset we have almost no errors in the recognition of B, B_T, M_P, P_H, S_B, W_B1, W_B2, and other activities. However, the recognition effect for E_H and L_H, E and W_T, W_TV, and W_L is a bit poor, which may be because these activities are represented by some of the same sensors compared to other activities, making them more difficult to recognize. For example, E_H and L_H both occur at the door, W_T and E both occur in the dining room, and W_TV and W_L both occur in the bedroom.

Compared with Tulum2010, the Cairo dataset has fewer activities. From Table 7, we can see that there are three misidentifications of N_W activity as B_T. This may be because the routes and the occurrence times of these three N_W are similar to B_T. However, it can be seen that the overall recognition effect of our method is still very good.

### 5.4. Parameter Selection

#### 5.4.1. Selection of *k*

Our *k*-value selection method is to use the elbow method. Its formula is shown in Equation (1). The core idea of the elbow method is: as the number of clusters *k* increases, the sample division will become more refined, and the degree of aggregation of each cluster will gradually increase, so the *SSE* will naturally become smaller. Moreover, when k is less than the number of true clusters, the increase of *k* will greatly make the degree of aggregation of each cluster increasing, so the decline of *SSE* will be quick. When *k* reaches the true number of clusters, the increase of the aggregation degree obtained by increasing *k* will be slow, so the decline of *SSE* will decrease sharply, and then gradually flatten as the value of *k* continues to increase. In other words, the relationship between *SSE* and *k* is the shape of an elbow, and the value of *k*, this elbow is the true cluster number of the data. Of course, this is why the method is called the elbow method.

**Figure 4:**
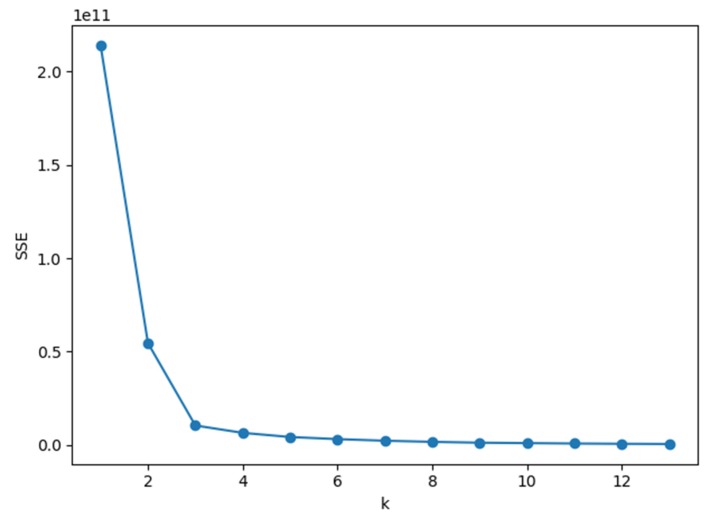
Relationship between *k* and sum of the squared errors (*SSE*) in Tulum2010.

Figure 4 shows the relationship between *k* and *SSE* in the Tulum2010 dataset. It can be seen that the curvature is the largest when *k* = 3, so we choose *k* = 3 as the optimal number of clusters.

#### 5.4.2. Selection of n and w_1_, w_2_ Values

**Figure 5:**
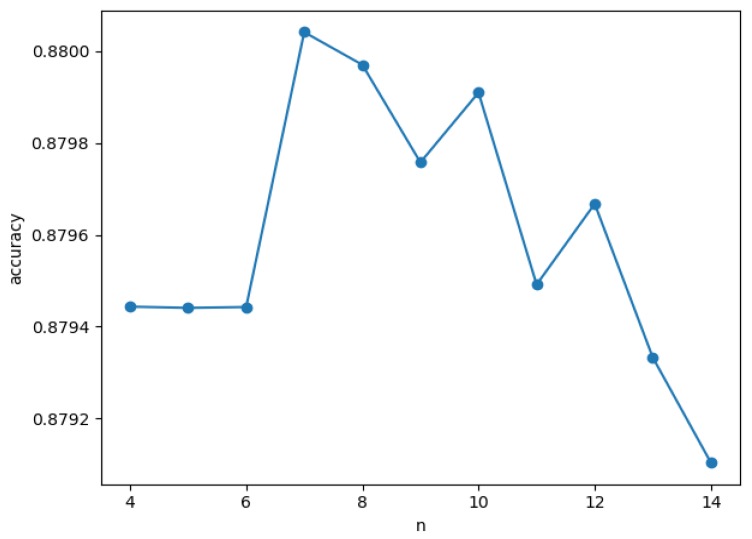
Relationship between *n* and *accuracy* when weight of ratio *(w*_1_) = 0.15 and *w*_2_ = 0.7.

We believe that the choice of *n* value should be less than the number of kinds of activities in the data set, which makes our results more accurate. Equation (2) gives a detailed similarity formula. For *w*_1_ and *w*_2_, we always keep ${2*w}_{1}\leq w_{2}$ and ${2*w}_{1}{+w}_{2}=1$. Figure 5 shows the relationship between n and accuracy when *w*_1_ = 0.15 and *w*_2_ = 0.7 in the Tulum2010 data set. As you can see, the fluctuation of accuracy is small.

## 6. Conclusions

This paper presents a daily activity recognition method based on time clustering for two residents in a smart home. First, noise reduction processing is performed on the features. Second, cluster is performed to separate activities that occur at the same space but at different times. Finally, a similarity matching formula based on Levenshtein Distance is proposed for daily activity recognition. The proposed method not only reduces the interference caused by the activities of different residents in daily life, but also separates the activities of residents at different times in the same space. We evaluated the proposed on two public datasets, Tulum2010. The results show that our method works well on large and small datasets.

## Figures and Tables

**Table 1:** A segment of activity records.

id	d	t	s	ss	ar	as
se_1_	2010-11-04	05:40:51.303739	M004	ON	Bed_to_Toilet	begin
se_2_	2010-11-04	05:40:52.342105	M005	OFF		
se_3_	2010-11-04	05:40:57.176409	M007	OFF		
se_4_	2010-11-04	05:40:57.941486	M001	OFF		
se_5_	2010-11-04	05:43:24.021475	M004	ON	Sleep	begin
se_6_	2010-11-04	05:43:26.273181	M004	OFF		
se_7_	2010-11-04	05:43:26.345503	M007	ON		
se_8_	2010-11-04	05:43:26.793102	M007	ON	Bed_to_Toilet	end
se_9_	2010-11-04	05:43:27.195347	M006	OFF		
se_10_	2010-11-04	05:43:27.787437	M007	ON		
se_11_	2010-11-04	05:43:29.711796	M005	ON		
se_12_	2010-11-04	05:43:30.279021	M004	OFF	Sleep	end

**Table 2:** Statistical information concerning datasets “Tulum2010” and “Cairo.”.

	Sensors	Activity Categories	Activity Instances	Residents	Measurement Time
Tulum2010	36 (2 categories)	14	7980	2	98 days
Cairo	32 (2 categories)	13	600	2	57 days

**Table 3:** Confusion matrix presenting number of true positives, true negatives, false positives, and false negatives for a 2-class classification problem.

		Actual Class	
		1	2
Predicted Class	1	TP(true positive)	FP(false positive)
	2	FN(false negative)	TN(true negative)

**Table 4:** Tulum2010 performance.

	Accuracy	Precision	Recall	F-Measure
KNN	77.10%	77.20%	77.10%	77.10%
LibSVM	72.40%	75.90%	72.40%	71.70%
SMO	54.20%	61.60%	54.20%	50.10%
NB	50.60%	65.50%	50.60%	52.60%
RIPPER	80.30%	80.50%	80.30%	80.30%
C4.5	80.90%	81.00%	80.90%	80.90%
RF	83.60%	83.50%	83.60%	83.40%
Our Method	88.10%	88.00%	88.10%	87.90%

**Table 5:** Cairo performance.

	Accuracy	Precision	Recall	F-Measure
KNN	80.20%	81.40%	80.20%	80.40%
LibSVM	30.30%	38.50%	30.30%	27.00%
SMO	71.70%	76.90%	71.70%	68.40%
NB	79.20%	81.70%	79.20%	79.50%
RIPPER	76.80%	78.30%	76.80%	76.90%
C4.5	83.00%	83.20%	83.00%	83.00%
RF	89.60%	89.90%	89.70%	89.70%
Our Method	92.00%	92.90%	92.00%	92.00%

**Table 6:** Confusion matrix for Tulum2010.

	B	B_T	E	E_H	L_H	M_P	P_H	S_B	W_TV	W_B1	W_B2	W_L	W_T	Y
**B**	80						1							
**B_T**		16					6				1			
**E**			62		1	3							27	
**E_H**				8	5				1		1			
**L_H**				4	9									2
**M_P**			5			192							1	
**P_H**	1	2					169							
**S_B**								36			2			
**W_TV**									278		1	45		
**W_B1**										94				
**W_B2**										1	267			
**W_L**									54			60		
**W_T**			19			2							136	
**Y**									3					2

**Table 7:** Confusion matrix for Cairo.

	B_T	B	D	L	L_H	L	N_W	R1_S	R1_W	W_O	R2_S	T_M	R2_W
**B_T**	6												
**B**		8			1								1
**D**			9										
**L**				1	1								
**L_H**					13					1			
**LUN**						8							
**N_W**	3						11						
**R1_S**							1	9					
**R1_W**	1								10				
**W_O**										10			
**R2_S**								1			10		
**T_M**												9	
**R2_W**													11
